# Expression of RING Finger Protein 38 in Serotonergic Neurons in the Brain of Nile Tilapia, *Oreochromis niloticus*

**DOI:** 10.3389/fnana.2018.00109

**Published:** 2018-12-06

**Authors:** Kai Lin Cham, Tomoko Soga, Ishwar S. Parhar

**Affiliations:** Brain Research Institute, School of Medicine and Health Sciences, Monash University Malaysia, Bandar Sunway, Malaysia

**Keywords:** E3 ubiquitin ligase, periventricular pretectal nuclei, raphe, brain, tilapia, teleost

## Abstract

Serotonin (5-hydroxytryptamine, 5-HT) is one of the major neurotransmitters, modulating diverse behaviours and physiological functions. Really interesting new gene (RING) finger protein 38 (RNF38) is an E3 ubiquitin ligase whose function remains unclear. A recent study has shown a possible regulatory relationship between RNF38 and the 5-HT system. Therefore, to gain insight into the role of RNF38 in the central 5-HT system, we identified the neuroanatomical location of 5-HT positive cells and investigated the relationship between RNF38 and the 5-HT system in the brain of the Nile tilapia, *Oreochromis niloticus*. Immunocytochemistry revealed three neuronal populations of 5-HT in the brain of tilapia; the paraventricular organ (PVO), the dorsal and ventral periventricular pretectal nuclei (PPd and PPv), and, the superior and inferior raphe (SR and IR). The 5-HT neuronal number was highest in the raphe (90.4 in SR, 284.6 in IR), followed by the pretectal area (22.3 in PPd, 209.8 in PPv). Double-label immunocytochemistry showed that the majority of 5-HT neurons express RNF38 nuclear proteins (66.5% in PPd; 77.9% in PPv; 35.7% in SR; 49.1% in IR). These findings suggest that RNF38 could be involved in E3 ubiquitination in the central 5-HT system.

## Introduction

The monoamine neurotransmitter serotonin (5-hydroxytryptamine, 5-HT) is one of the major neurotransmitters, modulating diverse behavioural and physiological functions such as mood, fear, aggression, appetite, sleep, memory and reproduction (Jacobs and Azmitia, 1992; Lucki, 1998; Charnay and Léger, 2010; Lillesaar, 2011). Furthermore, 5-HT has also been shown to regulate developmental processes such as cell migration, cell proliferation, neuronal differentiation, neurogenesis, synaptogenesis, and apoptosis (Azmitia, 2001; Gaspar et al., 2003; Cote et al., 2007; Pérez et al., 2013). The wide variety of 5-HT-mediated functions indicate an important role in the central nervous system, and dysfunction in the 5-HT function is often associated with several neuropathologic conditions such as anxiety, schizophrenia and depression (Charney et al., 1987; Owens and Nemeroff, 1994; Breier, 1995). Despite concerted efforts to characterise 5-HT’s multiple roles, the target brain regions responsible for its capacity to modulate behavioural and neuroendocrine responses, and the underlying mechanisms involved in these neurological disorders, remain poorly understood.

The central 5-HT system is well characterised in the brains of vertebrates. In all vertebrates studied, such as mammals (Dahlström and Fuxe, 1964; Steinbusch, 1981; Charnay and Léger, 2010), amphibians (Ueda et al., 1984; Clairambault et al., 1994), reptiles (Wolters et al., 1985; Ayala-Guerrero et al., 1991), birds (Challet et al., 1996) and fish (Meek and Joosten, 1989; Kaslin and Panula, 2001; Loveland et al., 2014), the main population of the 5-HT-containing neurons is restricted in the raphe nucleus in the hindbrain. These raphe 5-HT neurons can be further classified into the superior raphe (SR) and the inferior raphe (IR) subnuclei based on their neuroanatomical locations, projection areas, electrophysiological properties, molecular architectures and functional properties (Kirby et al., 2003; Abrams et al., 2004; Beck et al., 2004; Wylie et al., 2010; Gaspar and Lillesaar, 2012; Loveland et al., 2014). Studies have shown that 5-HT neurons in the SR give rise to ascending pathways that innervate most of the brain areas, whereas the IR provides descending pathways to the brain stem and spinal cord. In fish, 5-HT neurons are also found in the nucleus of the paraventricular organ (PVO) and periventricular pretectal nuclei (PP) (Kaslin and Panula, 2001; Loveland et al., 2014).

A recent study has shown that really interesting new gene (RING) finger protein 38 (RNF38) is modulated by the 5-HT system (Moriya et al., 2015). RNF38 is a member of the RING finger family, characterised by a RING domain and a zinc-binding motif. It possesses a RING-H2 domain (C_3_H_2_C_2_) and a coiled-coil domain, suggesting that it is involved in protein-protein/DNA interactions (Eisenberg et al., 2002; Moriya et al., 2015). The function of RNF38, to date, remains unclear. However, RING finger proteins have been shown to regulate various cellular processes such as the cell cycle (Gmachl et al., 2000; Jia et al., 2009), cell signalling (Vallabhapurapu et al., 2008), DNA repair (Gowen et al., 1998; Ulrich and Jentsch, 2000), vesicular trafficking (Srivastava et al., 2000; Matsuda et al., 2001), tumour suppression (Roperch et al., 1999; Sanada et al., 2009) and apoptosis (Hu and Yang, 2003; Kojima et al., 2005). *Rnf38* mRNA is found in the brain, spleen, liver, heart, testis, and placenta (Eisenberg et al., 2002), and its protein is a nuclear protein capable of mediating ubiquitination of the p53 tumour-suppressor protein (Sheren and Kassenbrock, 2013), thus acting as an E3 ubiquitin ligase. In the brain, *rnf38* mRNA and its protein are widely distributed, including the telencephalon, preoptic area, optic tectum, hypothalamus, cerebellum and the hindbrain in male tilapia (Cham et al., 2017). Furthermore, the same study showed that RNF38 protein is absent in astroglial cells, but is expressed in the nucleus of neuronal cells (Cham et al., 2017), suggesting that RNF38 may be involved in neuronal functions by interacting with various proteins. RNF38 is also linked to cancerous diseases and ageing (Fu et al., 2006; Nacheva et al., 2010; Sato et al., 2013; Xiong et al., 2018). For example, *rnf38* mRNA and protein levels are upregulated in non-small cell lung cancer (NSCLC), which in turn increases proliferation and the metastatic capacity of the cells (Xiong et al., 2018), whereas *rnf38* mRNA is increased in the hypothalamus during ageing (Fu et al., 2006).

The regulatory relationship between RNF38 and the 5-HT system remains to be elucidated. As an E3 ubiquitin ligase, RNF38 maintains cellular function by controlling the protein homeostasis within neurons via the ubiquitin proteasome system (UPS). To understand the relationship between RNF38 and 5-HT, we localised the distribution of RNF38 and the 5-HT neurons in the brain of *Oreochromis niloticus* using double-labelling immunocytochemistry.

## Materials and Methods

### Animals

Sexually matured male Nile tilapia (*O. niloticus*) (6 months old) were maintained under a controlled natural photo regime (14-h light; 10-h dark cycle) in standard fish tanks (size: 450 × 295 × 300 mm) equipped with a continuous supply of aerated freshwater at 28 ± 0.5°C. The fish were fed twice per day with commercial cichlid pellets (Star Feedmills, Selangor, Malaysia). All experimental procedures were performed under the guidelines of Monash University Animal Ethics Committee, AEC (MARP/2015/109).

### Localisation of 5-HT Neurons in the Brain

#### Brain Tissue Preparation for Immunocytochemistry

Male tilapia (*n* = 2) were anaesthetised using a 0.02% benzocaine solution (Sigma, St. Louis, MO, United States). The fish were then decapitated and the brains harvested. Gonads were examined macroscopically to confirm that the fish were sexually matured. The whole brain samples were then fixed in 4% paraformaldehyde in a 0.1 M phosphate buffer (pH 7.3) for 6 h at 4°C followed by cryoprotection in 20% sucrose in a 0.1 M phosphate buffer (pH 7.3). The cryoprotected whole brain samples were then embedded in frozen section compound (Leica, Wetzlar, Germany) and sectioned coronally at 15 μm using a cryostat (Leica CM1860). The sections were mounted onto silane-coated glass slides (Muto Pure Chemicals, Tokyo, Japan) and stored at -80°C until use for immunocytochemistry.

#### Immunocytochemistry

Sections were rehydrated in phosphate-buffered saline and a blocking solution containing 2% normal goat serum and 0.5% Triton-X was applied to each section. The sections were then incubated in primary polyclonal rabbit anti-5-HT antiserum (1:1000, 20080, RRID:AB_572263, Immunostar, Hudson, WI, United States) in a blocking solution in a closed moist chamber for 48 h at 4°C, followed by incubation in biotinylated anti-rabbit immunoglobulin IgG (1:200) for 30 min and avidin-biotinylated horseradish peroxidase complex (1:50) for 45 min at room temperature (PK-6101, RRID:AB_2336820, Vectastain ABC Elite Kit, Vector Laboratories, Burlingame, CA, United States). Antigen-antibody complexes were visualised with Alexa Fluor 488 Streptavidin (1:500; S32354, RRID:AB_2315383, Invitrogen Corporation, Carlsbad, CA, United States). The specificity of the 5-HT antibody was tested in tilapia brain in a previous study, where an absorption test with 5-HT bovine serum albumin (BSA) conjugate and 5-HT antibody omitted showed negative staining. (Loveland et al., 2014).

#### Image Analysis

Section images were captured using a MIRAX MIDI slide scanner (Carl Zeiss, Oberkochen, Germany) with an appropriate excitation filter for Alexa Fluor 488 and computer software (Pannoramic Scanner; 3DHISTECH, Budapest, Hungary). Nomenclature for the brain area was adopted from Parhar, 1990, Lillesaar (2011), and Loveland et al. (2014).

### Co-localisation of 5-HT Neurons With RNF38 Peptide in the Brain

#### Brain Tissue Preparation for Double-Labelling Immunocytochemistry

Male tilapia (*n* = 6) were anaesthetised using a 0.02% benzocaine solution (Sigma, St. Louis, MO, United States). Fish were then decapitated and the brains harvested. Gonads were examined macroscopically to confirm that the fish were sexually matured. The whole brain samples were then fixed in 4% paraformaldehyde in 0.1 M phosphate buffer (pH 7.3) for 6 h at 4°C followed by cryoprotection in 20% sucrose in 0.1 M phosphate buffer (pH 7.3). The cryoprotected brain samples were then embedded in frozen section compound (Leica, Wetzlar, Germany) and sectioned coronally at 15 μm using a cryostat (Leica CM1860). The coronal sections were divided into two sets and sections that were 30 μm apart were used for double-labelling immunocytochemistry. The sections were mounted onto silane-coated glass slides (Muto Pure Chemicals, Tokyo, Japan) and stored at -80°C until use.

#### Double-Labelling Immunocytochemistry of 5-HT Neurons With RNF38 Peptide

Sections were rehydrated in phosphate-buffered saline and a blocking solution containing 2% normal goat serum and 0.5% Triton-X was applied to each section. Primary polyclonal rabbit anti-RNF38 antiserum (1:200, ab121487, RRID:AB_11128227, Abcam, Cambridge, United Kingdom) in blocking solution was applied to all sections and the slides were incubated in a closed moist chamber for 48 h at 4°C. The sections then were visualised with goat anti-rabbit IgG secondary antibody, Alexa Fluor 594 conjugated (1.400, A11037, RRID:AB_2534095, Thermo Fisher Scientific, Waltham, MA, United States). Then, primary polyclonal rabbit anti-5HT antiserum (1:1000, 20080, RRID:AB_572263, Immunostar, Hudson, WI, United States) in a blocking solution was applied to all sections and the slides were incubated in a closed moist chamber for 48 h at 4°C. The sections were then incubated in biotinylated anti-rabbit immunoglobulin IgG (1:200) for 30 min and avidin-biotinylated horseradish peroxidase complex (1:50) for 45 min at room temperature (PK-6101, RRID:AB_2336820, Vectastain ABC Elite Kit, Vector Laboratories, Burlingame, CA, United States). Antigen-antibody complexes were visualised with Streptavidin, Alexa Fluor 488 conjugate (1:500; S32354, RRID:AB_2315383, Thermo Fisher Scientific, Waltham, MA, United States). The specificity of the RNF38 antibody was tested in the brain of tilapia in a previous study (Cham et al., 2017).

#### Image Analysis

Co-localisation of 5-HT neurons and RNF38 peptide was confirmed using the multiphoton and confocal fluorescence microscope (Leica TCS SP8 MP), equipped with Leica Application Suite X software (Leica, Wetzlar, Germany). Laser wavelengths of 488 and 552 nm were used for the excitation of fluorophores Alexa Fluor 488 and Alexa Fluor 594, respectively. Confocal images for analysis were laser-scanned with an XYZ resolution of 1024 × 1024 pixels that covered an area of 177.14 × 177.14 μm, using the ×25 objective. The images were collected as Z-stacks with a Z-step size of 0.57 μm, generating 56 frames. Z-series (frames) of confocal images were then used for 3D reconstruction. The 3D image rotation was carefully inspected to confirm expression of RNF38 in the nucleus of 5-HT neurons.

The total number of 5-HT neurons and the percentage of RNF38-positive 5-HT neurons were determined using scanned images of sections captured using a MIRAX MIDI slide scanner (Carl Zeiss, Oberkochen, Germany) with appropriate excitation filters for Alexa Fluor 594 and Alexa Fluor 488, and computer software (Pannoramic Scanner; 3DHISTECH, Budapest, Hungary). The area of interest for each subject was defined as the area between the first and the last sections that contained at least one 5-HT-immunoreactive cell. Cell counting of 5-HT neurons was performed manually based on observation from the scanned sectional images. A 5-HT neuron was counted as positive if the cell bodies with well- defined borders contained detectable green fluorescence labelling and the staining was even throughout the entire cell. The total number of the 5-HT neurons in PPd, PPv, SR and IF was calculated. The diameter of the 5-HT neurons in all 5-HT neuronal populations (PPd, PPv, SR and IF) was determined manually by using this equation: actual diameter = measured diameter on image / magnification. RNF38-immunopositive status within the 5-HT neuron was identified by manual observation from scanned sectional images. Green fluorescent labelled 5-HT neurons were considered RNF38-positive if their nucleus showed detectable red fluorescence immunolabelling and RNF38-negative if they showed no visible nucleus or a nucleus lacking RNF38 immunolabelling. Co-localisation was scored only if RNF38 was present in the nucleus of the 5-HT neurons. The co-localisation percentage of 5-HT neurons and RNF38 in the PPd, PPv, SR and IF with at least one co-localisation were calculated.

## Results

### Localisation of 5-HT Neurons in the Brain

**FIGURE 1:**
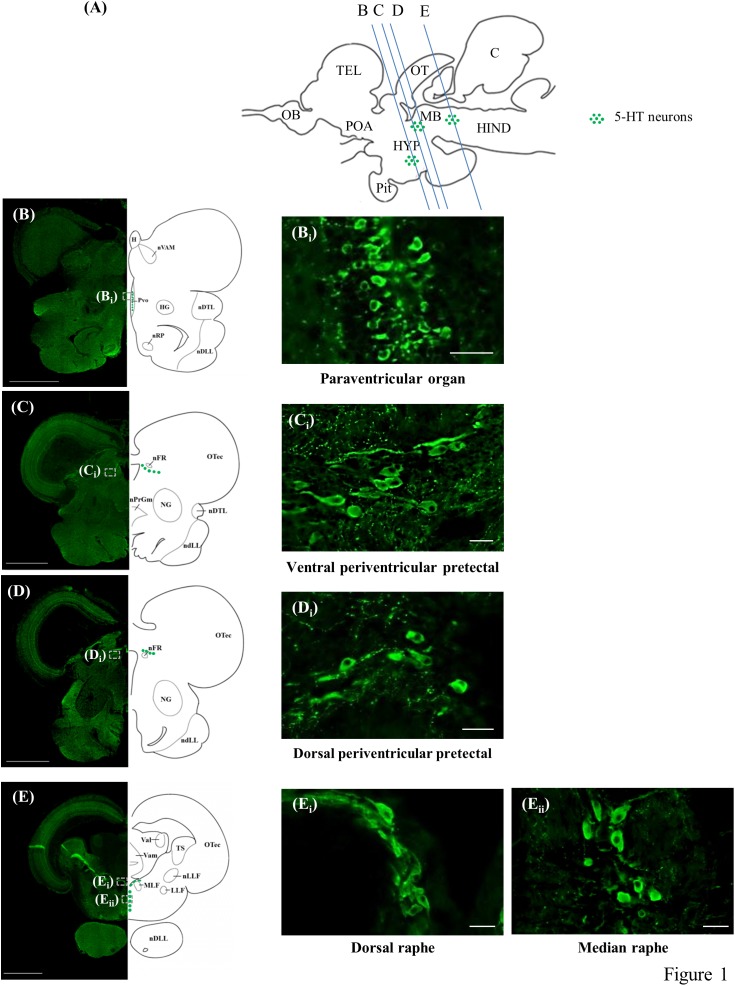
5-HT-immunoreactive neurons in the brain of tilapia. **(A)** Schematic sagittal diagram of tilapia brain with approximate locations of coronal sections **(B–E)**. **(B–E)** Representative low magnification photomicrographs of coronal sections expressing 5-HT immunoreactive neurons. Panels **(B_i_,C_i_,D_i_,E_i_**,**E_ii_)** show a higher magnification of the boxed area in panels **(B–E)**, respectively. Scale bars: **(B–E)** 1000 μm; **(B_i_,C_i_,D_i_,E_i_,E_ii_)** 25 μm.

The present study identified the distribution of 5-HT cell bodies in the brains of tilapia (Figures 1A–E_ii_). There were three neuronal populations of 5-HT in the tilapia brain; the nucleus of PVO, PP, and the raphe.

The periventricular groups within the posterior hypothalamus collectively constitute the PVO. In the PVO, the 5-HT neurons, with diameters of 5–6 μm, were relatively small and densely packed. The majority of the cells in this brain area appeared to have club-like apical dendritic processes extending and protruding into the third ventricle, possibly making contact with the cerebrospinal fluid (CSF) or lying within the ventricular wall (Figures 1B,B_i_). There was also a small group of 5-HT neurons (diameter of 5–6 μm) in the PVO that were globular with short processes and made no contact with the CSF in the third ventricle.

In the PP, the 5-HT neurons were classified into two groups, one situated dorsally of the fasciculus retroflexus (nFR) with another more ventrally. They constitute the dorsal periventricular pretectal nucleus (PPd) and ventral periventricular pretectal nucleus (PPv), respectively. In the anterior part of the PPv, the 5-HT cells, with diameters of 5–8 μm, were small globular cells clustering together, whereas 5-HT cell bodies in the posterior part of the PPv were relatively larger (diameter of 8–15 μm), with axons directed away from the third ventricle (Figures 1C,C_i_). The majority of these cells in the posterior area of the PPv were either globular or fusiform. In the PPd, the 5-HT cell bodies, with diameters of 8–12 μm, were either globular or fusiform (Figures 1D,D_i_).

In the raphe, 5-HT neurons were further subdivided into two subpopulations, one situated dorsally between the fasciculi longitudinalis medialis beneath the fourth ventricle and another more ventrally. They constitute the SR and IR, respectively. In the anterior part of the raphe population, the 5-HT neurons (diameter of 8–15 μm) in the SR were elongated and densely overlapped fusiform cells (Figures 1E,E_i_). In more posterior sections, 5-HT neurons (diameter of 8–15 μm) in the IR were either globular or fusiform cells arranged in a parallel manner along the medio-sagittal plane (Figures 1E–E_ii_).

### Total 5-HT Neuronal Number in the Brain

The 5-HT neuronal number was examined in four subregions of the brain; the PPd, PPv, SR and IR. The PVO population was omitted, as counting was difficult due to the morphology and distribution of the 5-HT neurons, where their cell bodies were small and densely packed together. Furthermore, PVO in teleost fish lacks *tph2* gene (Gaspar and Lillesaar, 2012), the key enzyme responsible for the biosynthesis of 5-HT in the brain. In the tilapia brain, the largest population of 5-HT cell bodies was in the raphe of the hindbrain. Male tilapia had an average of 90.4 ± 7.4 5-HT neurons in the SR and 284.6 ± 26.8 in the IR. In the midbrain, there were an average of 22.3 ± 3.3 5-HT neurons in the PPd and 209.8 ± 19.7 of 5-HT cells in the PPv (Table 1).

**Table 1:** Total 5-HT neuronal number and percentage of co-localisation of 5-HT neurons with RNF38 peptide in the dorsal periventricular pretectal nucleus, ventral periventricular pretectal nucleus, superior raphe and inferior raphe.

Brain area	Neuronal number	Percentage of co-localisation (%)
Dorsal periventricular pretectal	22.3 ± 3.3	66.5 ± 13.7
Ventral periventricular pretectal	209.8 ± 19.7	77.9 ± 7.2
Superior raphe	90.4 ± 7.4	35.7 ± 10.2
Inferior raphe	284.6 ± 26.8	49.1 ± 13.6


### Expression of RNF38 Peptide in 5-HT Neurons

**FIGURE 2:**
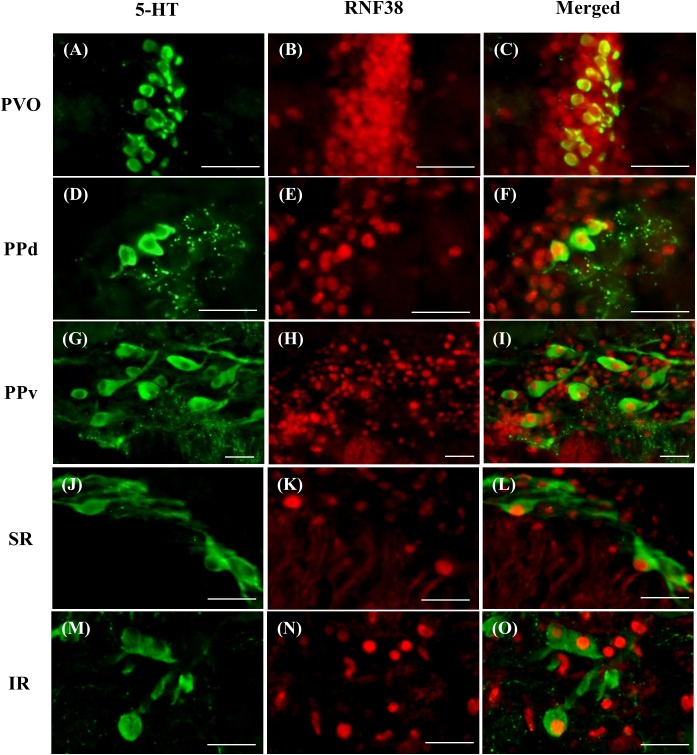
Double-labelling immunocytochemistry of 5-HT neurons (green, **A,D,G,J**,**M**) with RNF38 peptide (red, **B,E,H,K,N**) in paraventricular organ **(A–C)**, dorsal periventricular pretectal nucleus **(D–F)**, ventral periventricular pretectal nucleus **(G–I)**, superior raphe **(J–L)** and inferior raphe **(M–O).** Scale bars: **(A–O)** 25 μm.

Double-label immunocytochemistry showed that 5-HT neurons co-localised with RNF38 peptide in all brain areas where 5-HT neurons were localised (Figure 2). Midbrain periventricular pretectal had a higher percentage of 5-HT neurons expressing RNF38 peptide (66.5 ± 13.7% in PPd; 77.9 ± 7.2 % in PPv) than the hindbrain raphe 5-HT neurons (35.7 ± 10.2 % in SR; 49.1 ± 13.6 % in IR) (Table 1). The PVO population was omitted due to reasons mentioned in see Section “Total 5-HT neuronal number in the brain”.

## Discussion

### Localisation of 5-HT Neurons in the Brain

The present study examined the distribution of 5-HT neurons in the brain of the Nile tilapia. There were three neuronal populations of 5-HT in the tilapia brain: the nucleus of PVO, PP, and the raphe. The distribution of 5-HT-ir systems observed in the central nervous system of the Nile tilapia is generally in accordance with immunohistochemical studies of other teleost fish (Meek and Joosten, 1989; Batten et al., 1993; Kaslin and Panula, 2001; Loveland et al., 2014).

The present study showed 5-HT neurons in the PVO of the hypothalamus, consistent with studies in non-mammalian vertebrates, such as fish (Kah and Chambolle, 1983; Meek and Joosten, 1989; Kaslin and Panula, 2001; Loveland et al., 2014), amphibians (Sano et al., 1983; Ueda et al., 1984), reptiles (Sano et al., 1983; Ueda et al., 1983) and birds (Challet et al., 1996; Meneghelli et al., 2009). These densely packed 5-HT neurons in the PVO have processes that surround the blood vessels and extend into the third ventricle, suggesting that these cells can detect CSF and blood composition, and possibly secrete substances into the ventricular system and the circulation (Lillesaar, 2011). However, the functions of 5-HT neurons in the PVO remain largely unexplored. As the morphology of the 5-HT neurons in the PVO are very similar to the morphology of the CSF-contacting cells, it is possible that these 5-HT neurons are CSF-contacting cells that possess sensory receptors such as mechanoreceptors, chemoreceptors and photoreceptors (Vigh and Vigh-Teichmann, 1998). A recent study in the zebrafish showed that 5-HT in the PVO promotes neurogenesis of 5-HT neurons (Pérez et al., 2013), thus suggesting 5-HT is a potent regulator of neurogenesis. Besides, the 5-HT cells in the PVO have been shown to intermingle with dopaminergic and histaminergic cells (Kaslin and Panula, 2001; Filippi et al., 2010); however, the functional relationship between these monoaminergic systems remains to be elucidated. It has been proposed that the 5-HT observed in the PVO may be due to uptake from the third ventricle rather than interneuronal synthesis (Batten et al., 1993).

This study also revealed 5-HT cell bodies in the periventricular pretectal area of the brain. It is interesting to note that 5-HT neurons have been not been identified in the periventricular pretectal area in the brains of mammals, amphibians, reptiles or birds, suggesting that this is a fish-specific feature. Generally, this area is implicated in the modulation of the visuomotor behaviours and integration of multiple sensory modalities with visual input (Wullimann, 1997). In teleost, the optic tectum is densely innervated by 5-HT fibres (Batten et al., 1993; Kaslin and Panula, 2001) although it does not receive innervation from the raphe 5-HT neurons (Lillesaar et al., 2009). Therefore, it is highly possible that the 5-HT neuronal population in the pretectal area is the source of 5-HT innervation to the optic tectum. Therefore, 5-HT in the pretectal area may be involved in the regulation of optic tectum to coordinate the orientation of the eyes, body and tail movements, feeding and escape response, and motor movements (Meek, 1983; Salas et al., 1997; Herrero et al., 1998; Nevin et al., 2010).

The current study showed that the largest population of 5-HT neurons is in the raphe. The raphe 5-HT population is present in almost all vertebrates, such as mammals (Dahlström and Fuxe, 1964; Steinbusch, 1981; Charnay and Léger, 2010), amphibians (Ueda et al., 1984; Clairambault et al., 1994), reptiles (Wolters et al., 1985; Ayala-Guerrero et al., 1991), birds (Challet et al., 1996) and fish (Meek and Joosten, 1989; Kaslin and Panula, 2001; Loveland et al., 2014), indicating that the 5-HT neuronal population in the raphe is the most well-conserved 5-HT population, thus suggesting an important function in the central nervous system. In mammals, raphe 5-HT is subdivided into nine distinct subpopulations (B1–B9), where each subpopulation corresponds to specific brainstem nuclei (B1, raphe pallidus; B2, raphe obscurus; B3, raphe magnus; B4, dorsal to prepositus hypoglossi; B5, raphe pontis; B6, caudal part of raphe dorsalis; B7, raphe dorsalis; B8, centralis; B9, the supralemniscal nucleus) (Steinbusch, 1981; Gaspar and Lillesaar, 2012). However, raphe 5-HT cells in the fish appear to be rather scattered, therefore it is difficult to assign them to neuronal populations homologous to the nine 5-HT subpopulations as identified in mammals. Generally, in fish, raphe 5-HT is classified into SR and IR, where SR 5-HT cells located dorsally between the fasciculi longitudinalis medialis and IR 5-HT cells are located more ventrally. Apart from neuroanotamical locations, 5-HT neurons in the SR and the IR can also be distinguished based on the regions to which they project, electrophysiological properties, molecular architectures and functional properties (Kirby et al., 2003; Abrams et al., 2004; Beck et al., 2004; Wylie et al., 2010). Similar to mammals, in the zebrafish, SR provides ascending pathways which innervate most of the brain areas, whereas the IR provides descending pathways to the brain stem and the spinal cord (Lillesaar, 2011). The extensive projections of raphe 5-HT neurons to almost all the brain areas indicate widespread influences of 5-HT on behavioural and physiological functions. For example, ascending 5-HT neuronal projections from the raphe to the telencephalon modulates schooling, courtship and sexual and aggressive behaviours (Noble and Borne, 1941; de Bruin, 1980), and to the POA modulates reproduction and sexual behaviour (Verma et al., 1989; Hull et al., 2004). Descending 5-HT neuronal projections from the raphe to the brain stem and spinal cord are important for the mediation of sensory, somatic motor, autonomic and reflex control (Bowker et al., 1983).

### 5-HT Neurons Express RNF38 Peptide

5-HT modulates diverse behavioural and physiological functions such as mood, fear, aggression, appetite, sleep, memory and reproduction (Jacobs and Azmitia, 1992; Lucki, 1998; Charnay and Léger, 2010; Lillesaar, 2011). 5-HT has also been shown to regulate developmental processes such as cell migration, cell proliferation, neuronal differentiation, neurogenesis, synaptogenesis and apoptosis (Azmitia, 2001; Gaspar et al., 2003; Cote et al., 2007; Pérez et al., 2013). The presence of RNF38 in 5-HT neurons suggests that RNF38 might be involved in the regulation of these behavioural, physiological and developmental functions by modulating the 5-HT system.

To date, the functions of RNF38 remain unclear. A recent study has shown that RNF38 exhibits E2-dependent E3 ubiquitin ligase activity (Sheren and Kassenbrock, 2013; Buetow et al., 2015), indicating that RNF38 is involved in the ubiquitin proteosome system (UPS), which governs protein homeostasis within neurons by the continuous degradation of intracellular proteins by proteosomes. Several E3 ubiquitin ligases have been shown to regulate neuronal properties such as neurogenesis (Tuoc and Stoykova, 2008; Westbrook et al., 2008), neuronal growth and development (Tursun et al., 2005), synaptogenesis (DiAntonio et al., 2001) and synaptic plasticity (van Roessel et al., 2004) via the UPS. Therefore, as an E3 ubiquitin ligase, RNF38 may play a critical role in maintaining neuronal structures or regulating neuronal functions of the 5-HT system via the UPS.

A similar E3 ubiquitin ligase RINES/RNF180 has shown prominent functions in the modulation of the 5-HT system. RINES/RNF180 mediates ubiquitination of monoamine oxidase (MAO-A), a 5-HT-metabolising enzyme which plays a critical role in emotional and social behaviour (Cases et al., 1995; Deckert et al., 1999; Schulze et al., 2000; Bortolato et al., 2011). A recent study has shown that serotonin transporter (SERT) protein, a carrier protein, is ubiquitinated and degraded by proteasome (Mouri et al., 2016), but the ubiquitin ligase responsible for the ubiquitination of SERT protein remains unknown. Although E3 ubiquitin ligase confers substrate specificity, each individual E3 ubiquitin ligase is capable of recruiting more than one substrate and, similarly, the same substrate may be targeted by more than one E3 ubiquitin ligase (Nishitani et al., 2006; Stegmüller and Bonni, 2010). Thus, it is plausible that RNF38 can regulate 5-HT neuronal activity by targeting 5-HT-associated molecules such as MAO-A and SERT for ubiquitin-mediated degradation by proteasomes; this represents a possibility that needs to be investigated.

Since the deregulation of UPS is suggested to be involved in social behaviour and neurological disorders such as depression, the identification of RNF38 ubiquitin ligase pathways that control the 5-HT system could advance our understanding in these neurological disorders.

## Conclusion

In summary, we have identified the localisation of three 5-HT neuronal populations in the brain of tilapia; PVO, PP, and raphe. To our knowledge, this is the first evidence that RNF38 peptide is expressed in the nucleus of 5-HT neurons, suggesting that RNF38 may interact with cellular proteins and modulate cellular functions in different 5-HT neuronal populations.

## Author Contributions

KC conducted all the research experiments. TS confirmed all the data. IP and TS designed the experiments and edited the manuscript.

## Conflict of Interest Statement

The authors declare that the research was conducted in the absence of any commercial or financial relationships that could be construed as a potential conflict of interest.
